# Cantilever bending based on humidity-actuated mesoporous silica/silicon bilayers

**DOI:** 10.3762/bjnano.7.56

**Published:** 2016-04-28

**Authors:** Christian Ganser, Gerhard Fritz-Popovski, Roland Morak, Parvin Sharifi, Benedetta Marmiroli, Barbara Sartori, Heinz Amenitsch, Thomas Griesser, Christian Teichert, Oskar Paris

**Affiliations:** 1 Institute of Physics, Montanuniversitaet Leoben, Austria; 2 Max-Planck-Institut für Kohlenforschung, Mülheim an der Ruhr, Germany; 3 Institute of Inorganic Chemistry, Graz University of Technology, Austria; 4 Chair of Chemistry of Polymeric Materials, Montanuniversitaet Leoben, Austria

**Keywords:** AFM cantilever, bilayer bending, grazing incidence small-angle X-ray scattering (GISAXS), mesoporous film, sorption-induced deformation

## Abstract

We use a soft templating approach in combination with evaporation induced self-assembly to prepare mesoporous films containing cylindrical pores with elliptical cross-section on an ordered pore lattice. The film is deposited on silicon-based commercial atomic force microscope (AFM) cantilevers using dip coating. This bilayer cantilever is mounted in a humidity controlled AFM, and its deflection is measured as a function of relative humidity. We also investigate a similar film on bulk silicon substrate using grazing-incidence small-angle X-ray scattering (GISAXS), in order to determine nanostructural parameters of the film as well as the water-sorption-induced deformation of the ordered mesopore lattice. The strain of the mesoporous layer is related to the cantilever deflection using simple bilayer bending theory. We also develop a simple quantitative model for cantilever deflection which only requires cantilever geometry and nanostructural parameters of the porous layer as input parameters.

## Introduction

Because the bending of a microcantilever can be measured with extremely high accuracy, sensors utilizing this principle are in focus of recent research. Such sensors are able, for instance, to detect molecules adsorbing on the cantilever surface by simply reading out the deflection of a chemically modified cantilever [1]. In order to differentiate a spectrum of molecules, cantilever arrays were used to create an artificial “chemical nose”, leading to sensor systems which are able to detect diabetes in the human breath [2] or cancer [3–4]. The measured deflection is usually very small owing to only very few molecules reaching the surface of the cantilever. In order to use similar principles for actuation purposes, a larger deflection of the cantilever must be reached. One strategy is to increase the number of adsorbed molecules by increasing the total interaction area of molecules with the surface. This leads to the concept of using bilayer structures, with one of the layers having a large accessible (internal) surface area. In the natural world of plants for instance, humidity-induced bending of bilayer structures is frequently used for actuation purposes. Prominent examples are the opening of tree cones [5], or the complex movement of the dispersal units of wild wheat [6] and ice plants [7]. In all these systems the movement is caused by the bending of bilayer structures that swell anisotropically and differently between the two layers upon the change of relative humidity (RH) [8]. This bilayer principle has been used to build simple artificial actuators based on the swelling of paper–plastic bilayers [9], and also more general routes have been proposed to create directional movement by using swelling of polymers in combination with controllable anisotropy [10]. However, to our knowledge such concepts have not been used so far to create ceramic or metallic bilayer structures, which would have the advantage of their applicability at higher temperatures and in harsh environments. The potential of mesoporous “hard” materials such as nanoporous gold [11] or mesoporous silica [12] for such purposes has recently been highlighted, where the basic idea is to utilize the adsorption-induced deformation of highly porous materials. The simplest way of fabricating an actuator based on such materials would be a bilayer consisting of a thin bulk substrate covered by a film of the mesoporous material. The change of interfacial energies (i.e., solid–liquid and liquid–gas interfaces) during adsorption of a gas into the pore space will lead to a deformation of the porous part of the bilayer [13]. Since the substrate layer is expected to deform considerably less due to its much lower accessible surface area, this will result in a bending movement similar to a bimetal strip in response to a temperature change [9].

Here, we demonstrate for the first time the fabrication and humidity-controlled actuation of a microcantilever based on a mesoporous silica/nonporous silicon bilayer using a commercial AFM cantilever as a substrate. The simplicity and versatility of the approach is promising for applications in several fields where similar systems based on swellable polymers are not applicable.

## Experimental

### Sample preparation

AFM cantilevers and silicon wafers were coated with a mesoporous silica film using evaporation-induced self-assembly [14–16]. We employed tipless NSG30 cantilevers from NT-MDT (about 130 µm long, 45 µm wide, and about 4.5 µm thick) with a gold reflective coating on one side. The cantilevers were immersed in 2 M NaOH aqueous solution for 40 min, rinsed with ethanol and dried in a flow of inert gas (N_2_ or CO_2_). In the next step, the gold coating of the cantilever was hydrophobized using self-assembled monolayers (SAM) of dodecanethiol (≥98%, 471364-100ML, Aldrich) in a 1 mM solution in ethanol for 8 h. This hydrophobization was necessary to deposit silica only on the non-hydrophobic side of the cantilever and so that its reflective coating could still be used. The precursor solution for the mesoporous silica coating was prepared in a three-step process. First, 1.75 g of tetraethyl orthosilicate (TEOS) and 1.25 g of ethanol was mixed with 1.25 g of aqueous 10 M HCl solution and was stirred for 1 h. Second, 580 mg of the block copolymer P123 (435465-250ML, Aldrich) were dissolved in 26.85 g ethanol and stirred for 1 h, followed by adding the first solution. In the third step, 1.9 g of aqueous 1 M HCl solution was added to the mixture prepared in the second step. This final precursor solution was then stirred for 2 h. The precursor solution was deposited on the cantilever by dip coating with a withdrawal speed of 2 mm/s. After letting the film rest at room temperature for one day, the sample was calcined at a temperature of 450 °C. The temperature was increased by 1 °C/min, held at 450 °C for 2 h, and cooled down to room temperature at the same rate. The silicon wafers were prepared with the identical protocol as the cantilever samples, only the hydrophobization step with the SAMs was skipped.

### AFM

**Figure 1:**
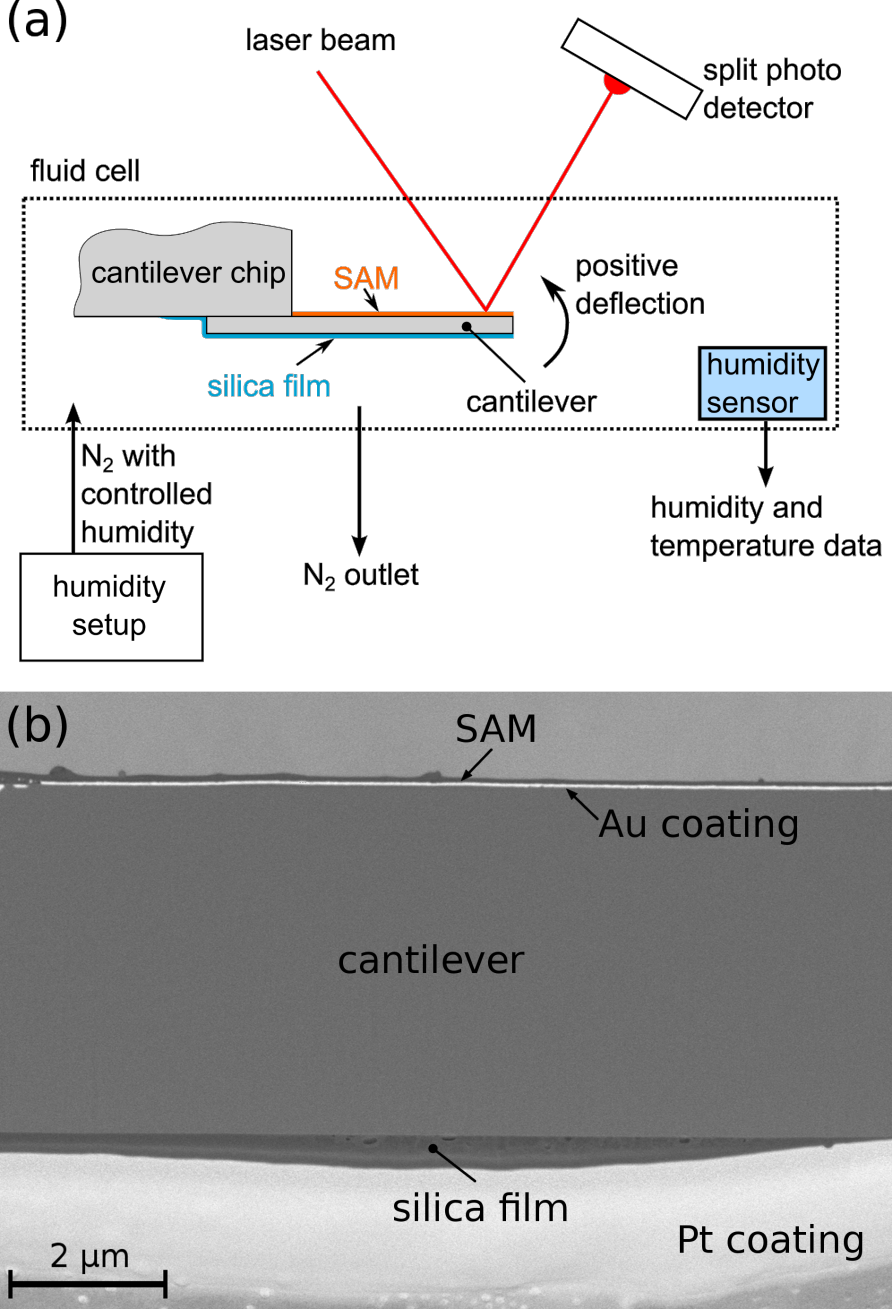
(a) Scheme of an AFM cantilever coated with a porous silica film in the fluid cell with deflection readout. (b) Backscattered electron SEM image of the cross section of a silica-coated cantilever.

The atomic force microscope in use was an Asylum Research MFP-3D, of which only the cantilever deflection was read-out and recorded as a function of the time. The deflection read-out was performed by the light-beam method, where a laser beam is reflected from the gold coating of the cantilever onto a split photodiode (Figure 1a). The control of relative humidity was achieved with a custom-built setup where a nitrogen stream is saturated with water in a bubbler and mixed with a dry nitrogen stream [17]. By manually adjusting the flow rates of the two streams, the relative humidity can be continuously controlled. The AFM cantilever was mounted inside a closed commercial fluid cell with a volume of about 10 mL. In addition, a Sensirion SHT21 sensor, which records the relative humidity as well as the temperature, is mounted in the fluid cell. Relative humidity and temperature were recorded as a function of time. This allows for correlating the cantilever deflection with the relative humidity. To properly quantify the cantilever response to the change in relative humidity as a deflection, the cantilever needs to be calibrated. The thermal sweep method [18] was used to calibrate the cantilevers and was performed directly before every measurement. In order to start every experiment under the same conditions, the first step was to reduce RH to the lowest possible value (around 5%). The cantilever was left in these conditions for several hours to equilibrate RH and the cantilever deflection to a stationary value. From this point, the adsorption curve followed by the desorption curve were recorded stepwise with 30–45 min equilibration time before reading out the cantilever deflection for each step. A cross section of the AFM cantilever after the actuation experiment was prepared with focused ion beam (FIB) cutting using an AURIGA Crossbeam Workstation (Zeiss). Scanning electron microscopy (SEM) images of the sample cross-section were taken with the same instrument with the electron microscope operated at a voltage of 2 keV.

### GISAXS: measurements and data evaluation

GISAXS measurements of the films prepared on Si wafers were performed at the Austrian SAXS beamline at the synchrotron radiation source ELETTRA in Trieste, Italy [19]. The wavelength was λ = 0.154 nm and 2D GISAXS data were collected with a Pilatus 1M detector (Dectris, Baden Switzerland) at a distance of 1502 mm. For the in situ water sorption measurements, a custom-built humidity cell with Kapton X-ray windows was used. The working principle is very similar to the humidity chamber for AFM described above with a humidity range between about 10 and 90% RH (for more information on the humidity cell, see [20]). In contrast to the AFM measurements, RH was changed continuously during the GISAXS measurements with a nominal rate of 0.5% per minute. The GISAXS measurement time was one minute per frame. After reaching the highest humidity (typically 92–93%), the sample was kept at this RH for 30 min and a desorption cycle with the same rate of RH and the same measurement time was started. Silver behenate was mounted as a standard on the sample cell.

Instabilities of the primary beam position were corrected by computing the center of the silver behenate rings for each frame. Changes of the incidence angle were monitored by the position of the specular reflection. Peak positions were computed as centers of gravity in cuts parallel and normal to the wafer surface after subtraction of a linear background. The obtained positions were corrected for refraction effects [21].

## Results and Discussion

### Cantilever deflection

In Figure 1a, the scheme of the coated cantilever is shown and in Figure 1b, the successful coating is demonstrated by an SEM image of the cantilever cross-section after the actuation experiment within the AFM. The different materials can easily be distinguished by the backscattered electron contrast, revealing bright contrast for the Au coating at the top, and the additional Pt coating required for clean FIB cutting at the bottom of the image. A compact silica film with only few defects formed on the bottom (hydrophilic) side of the silicon substrate. This film is thickest (approx. 500 nm) in the center of the cantilever cross section and becomes thinner towards the edges, with an average thickness of roughly 300 nm. The top (hydrophobized) side shows that a very thin silica film was formed also on the Au coating, which means that the hydrophobization with SAMs did not work as perfectly as planned. However, the mesoporous silica film is much thicker (by a factor of 8–10) at the bottom side without the SAMs. Therefore, the top silica film was not taken further into account.

**Figure 2:**
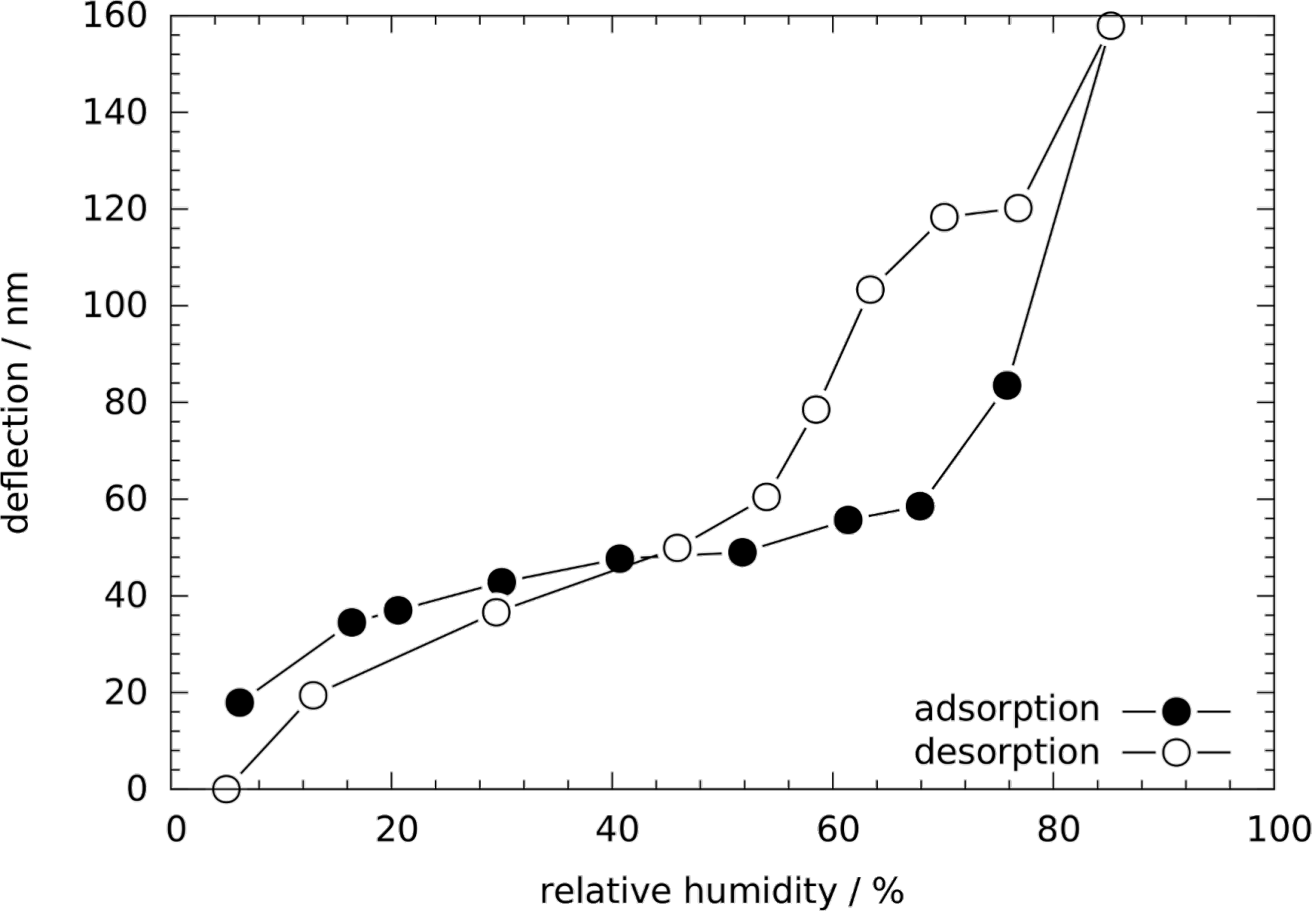
Cantilever deflection as a function of the relative humidity.

When the cantilever was placed in the AFM, a clear response to a change in relative humidity was evident. This response is shown as the cantilever deflection in Figure 2 during an adsorption cycle followed by a desorption cycle. The difference between minimum and maximum deflection was found to be about 140 nm, with a slight offset (about 20 nm) between the deflections before adsorption and after desorption. Such an offset points to an only partially reversible process and perhaps also to slight drift problems. A clear hysteresis is evident between the adsorption and desorption curves above RH = 50%. This hysteresis in cantilever deflection agrees with the hysteresis seen in water sorption isotherms measured for similar materials, indicating the different pressures for capillary condensation during adsorption and capillary evaporation during desorption, respectively, in pores with quite monodisperse size distributions [22]. It is well known that adsorption and desorption of fluids leads to a non-monotonous deformation of mesoporous materials [12–13]. In particular the so-called strain-isotherm, i.e., the deformation of the porous material as a function of relative fluid pressure, exhibits a similar hysteresis as the sorption isotherm. The observed cantilever bending is most likely induced by the sorption-induced deformation of the mesoporous film. Therefore, we attribute the strong change in the slope of the curves seen in Figure 2 (about 70% RH for adsorption and about 55% RH for desorption) to the transition from film formation on the pore walls to the spontaneous filling/emptying (capillary condensation/evaporation) of the pores with water.

Because the change of the relative humidity of air will also change its index of refraction (IOR), *n*, and the deflection read-out was done via a laser beam reflected from the cantilever, we have checked whether this can influence our results. The IOR change between dry air and air fully saturated with water is given by Δ*n* ≈ 1·10^−4^ [23]. Taking this into account, a detected deflection of 200 nm would be artificially increased due to the IOR change by only 0.2%, which can be neglected. This fact was also verified experimentally by placing an uncoated, pristine cantilever in the fluid cell and increasing the relative humidity from 5 to 90% while recording the cantilever deflection. In this experiment, only a drift of the deflection of approximately 5 nm was found but no significant change in deflection. Therefore, the data shown in Figure 2 reflect the cantilever deflection due to the change in humidity with an accuracy better than 5%.

### Characterization of mesoporous films

**Figure 3:**
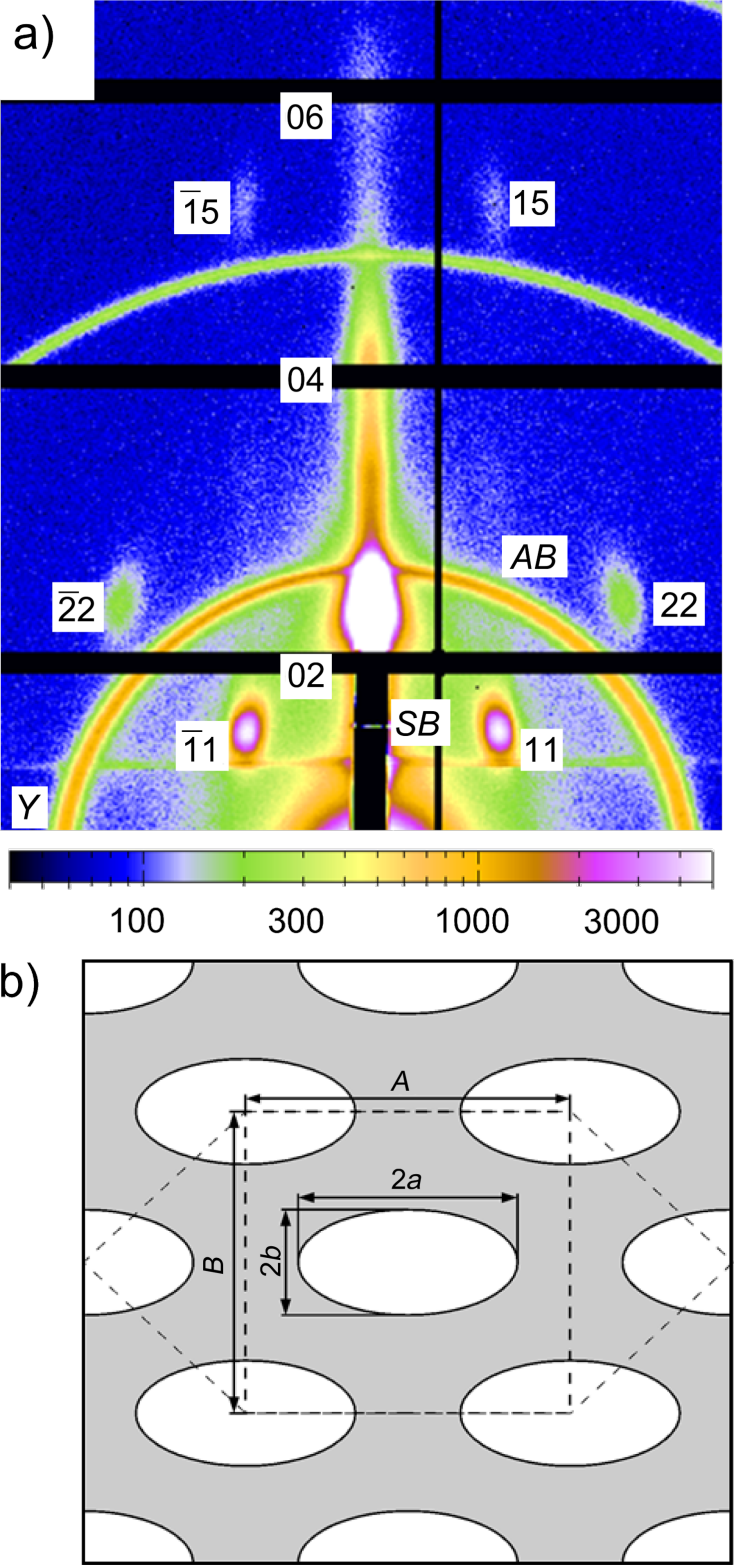
(a) GISAXS pattern of mesoporous silica film on a silicon substrate. The rings (marked AB) are from a silver behenate internal standard, and the black lines are dead areas of the detector. The numbers denote the Miller indices of the reflections of a centered rectangular 2D lattice, *Y* is the Yoneda peak, and *SB* the specular reflected beam. The grazing angle was 0.384°. (b) Unit cell of the 2D pore lattice reconstructed from the positions of the GISAXS reflections with lattice parameter *A* = 15.08 nm and *B* = 14.01 nm, and pore size parameters estimated from the intensities of the reflections assuming an elliptical cross-section of the cylindrical pores; 2*a* = 10.2 nm and 2*b* = 4.90 nm. Please note that the cylindrical pores lie preferentially within the film plane.

A mesoporous film on silicon substrate was prepared in the same way as the film on AFM cantilever to obtain a macroscopically extended film for the nanostructure characterization using GISAXS. The nanostructure of this material consists of quite monodisperse cylindrical pores ordered on 2D pore lattice [24]. The GISAXS pattern of the corresponding sample shown in Figure 3 reveals sharp Bragg reflections from this pore lattice. The fact that these reflections are not azimuthally smeared means that the long axis of the cylindrical pores expected from this type of materials shows a high degree of alignment to the substrate. The pattern reveals fiber symmetry around the film normal, which means that the orientation of the cylinder axes within the plane of the film is random. From the positions of the diffraction peaks, the lattice symmetry can easily be deduced. As reported already in earlier work [24], this symmetry deviates considerably from the 2D hexagonal lattice found for powder SBA-15 materials [25]. This is understood by the fact that the silicon substrate induces a strong mechanical constraint to the silica film. For an infinitely extended thin film tightly attached to a rigid substrate, film shrinkage would only be possible perpendicular to the substrate, but not parallel to it. This was demonstrated recently on similar samples for the water-sorption-induced deformation of the silica films [20]. Even much stronger deformation is expected during drying and calcination of the as synthesized films, causing the pore lattice to strongly shrink in the direction perpendicular to the substrate, while it is kept almost constant in the direction parallel to the substrate. As a consequence, the originally hexagonal pore lattice is strongly deformed. Figure 3b illustrates this by showing the centered rectangular unit cell of the 2D pore lattice extracted from the GISAXS data. While for a hexagonal symmetry the lattice parameters *A* and *B* would be related by 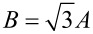, Figure 3b rather suggests *B* ≈ *A*. This is of course only possible, if also the cross-section of the cylindrical pores changes from originally circular to elliptical. A detailed analysis of the intensities of the individual reflections in the GISAXS pattern can be used to obtain an estimate for the two half axes *a* and *b* of the elliptical pore cross-section, which are given in Figure 3b. Together with the lattice parameters *A* and *B* of the centered rectangular unit cell, the pore volume fraction (porosity) of the film can easily be calculated by 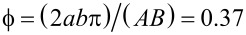. Moreover, the mean curvature of the pore cross-section can be determined by κ = 0.5(1/*a* + 1/*b*)*,* which leads to a mean radius of curvature *r* = 1/κ = 3.31 nm [26].

**Figure 4:**
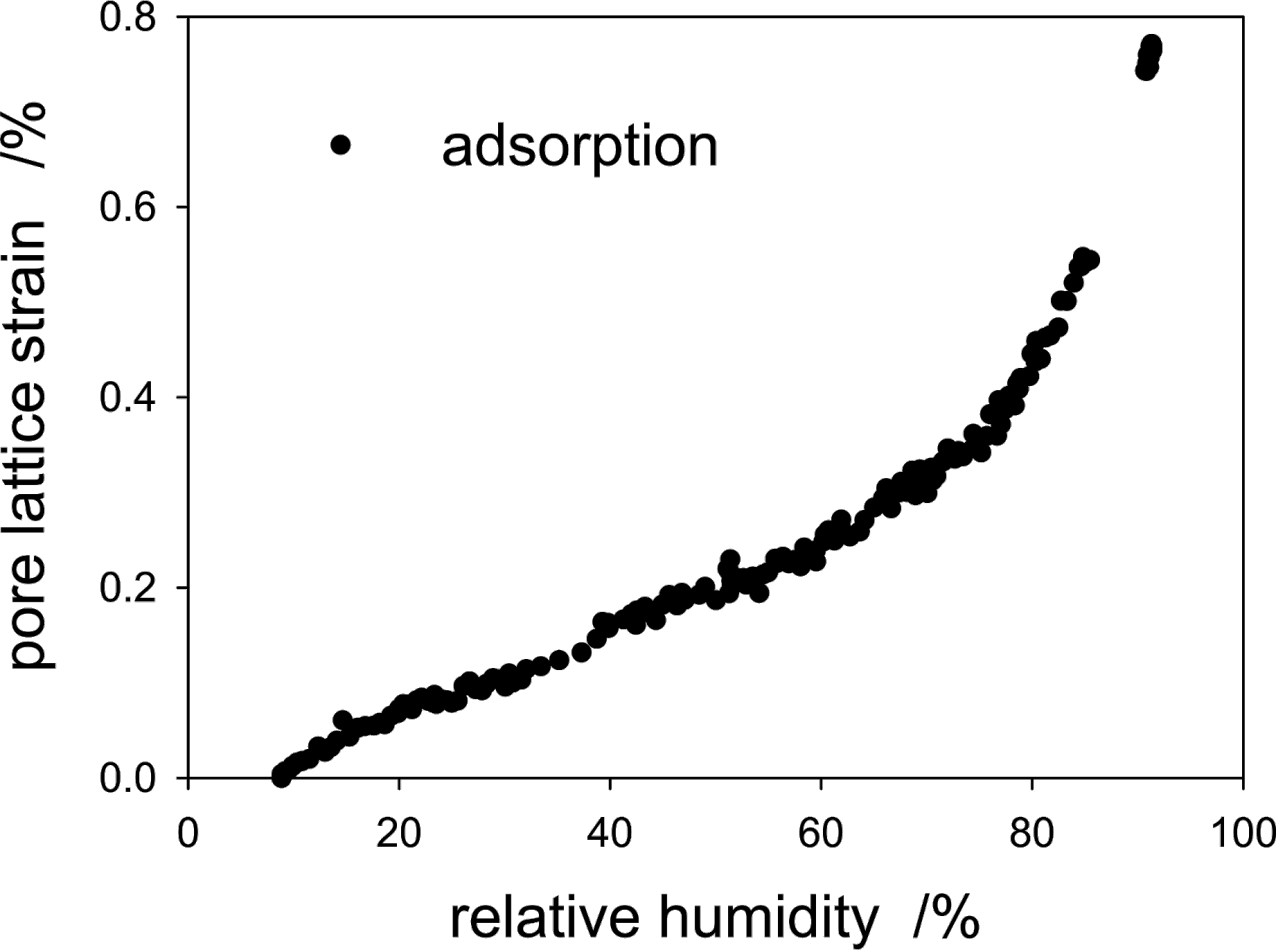
Strain isotherm from in situ GISAXS derived from the shift of the out-of-plane 02 reflection in Figure 3a. Only the adsorption branch could be measured reliably with the humidity setup used.

GISAXS patterns were collected in situ while changing the RH. From the shift of the Bragg peaks as a function of the RH, the pore lattice strain can be obtained [12,27–28]. Similar to our previous work [20], this strain was found to be much smaller in-plane (i.e., parallel to the surface) as compared to the out-of-plane direction perpendicular to the surface. Figure 4 shows the pore lattice strain obtained from the shift of the 02 reflection as a function of the RH during adsorption. Unfortunately, the corresponding desorption run could not be evaluated due to problems with humidity equilibration within the restricted time available for the synchrotron radiation experiment. Nonetheless, for the adsorption branch the pore lattice strain ε measured at the level of the individual pores can now be compared with the macroscopic cantilever deflection δ shown in Figure 2. Both quantities increase more gently at low RH, and become considerably steeper above 70%, where the onset of capillary condensation is expected. Indeed, the shape of the cantilever deflection vs RH curve in Figure 2 is very similar to the strain vs RH curve of Figure 4, suggesting a linear relationship between these two quantities.

### Quantitative description of cantilever deflection

In the following we attempt to describe the measured deflection of the cantilever on a quantitative basis. For this, we consider only the maximum measured deflection between the “dry” sample (i.e., below 10% humidity) and the “wet” sample. Since the deflection of the cantilever was only measured up to 85% RH, we take the same RH interval for the measured pore lattice strain. Table 1 summarizes the parameters used in the following.

**Table 1:** Maximum cantilever deflection δ and maximum pore lattice strain ε, both at RH = 85%; pore mean radius of curvature *r*, pore volume fraction 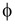, effective cantilever length *l,* silicon substrate thickness *d*_S_, average film thickness *d*_F_, Young’s modulus of silica *E*_S_, and change of surface energy Δγ of silica upon full wetting with water.

	δ (nm)	ε (%)	*r* (nm)	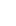 (%)	*l* (μm)	*d*_S_ (μm)	*d*_F_ (μm)	*E*_S_ (GPa)	Δγ (J/m^2^)

value	140	0. 55	3.31	37	130	4.5	0.3	130	0.12
reference	AFM	GISAXS	GISAXS	GISAXS	SEM	SEM	SEM	[29]	[30]

The linear deflection δ of a bilayer cantilever (i.e., a film *F* on a substrate *S*) with length *l* and total thickness *d* = *d*_F_ + *d*_S_ clamped at one end is given for small deflections (δ << bending radius) by [9]

[1]
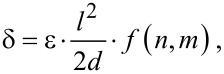


where ε is the strain difference (i.e., the difference in the relative expansion) between the two layers. The function *f* = 6(1 + *m*)^2^/[3(1 + *m*)^2^ + (1 + *mn*)(*m*^2^ + 1/*mn*)] depends on the thickness ratio *n* = *d*_F_/*d*_S_ of the two layers, and on the ratio of their Young’s moduli *m* = *E*_F_/*E*_S_ [31]. In our case both ratios are much smaller than one and *f* reduces to *f* = 6*mn*, which after approximating *d*_S_ by *d* leads to a very simple expression for Equation 1.

[2]
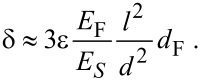


Since the silicon substrate can be regarded as rigid, ε is given by the strain in the silica film, which was measured experimentally by GISAXS (Table 1). Equation 2 confirms the linear relationship between the sorption-induced strain and the cantilever deflection. If we have a reliable estimate for the Young’s modulus *E*_F_ of the film, the expected deflection can be predicted from the experimentally measured strain (see Figure 4). However, *E*_F_ is not known experimentally for our film. On the other hand, if we know both, the cantilever deflection and the sorption-induced strain from experiment, Equation 2 can be used to estimate the Young’s modulus of the porous film. Inserting the experimental values of δ and ε together with the other known parameters (Table 1) we obtain *E*_F_ ≈ 5.0 GPa. Literature reports values of the order of 30–40 GPa for the bulk Young’s modulus 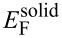 in nanostructured amorphous silica systems [32–33]. If we assume quadratic scaling of the Young’s modulus with density 
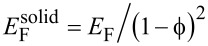
 [34], *E*_F_ would be expected to be of the order of 12–16 GPa, which is higher by a factor of 2–3 than the value obtained from Equation 2. This discrepancy can be resolved when considering that the pore volume fraction obtained from GISAXS (Table 1) does not include any micropores (i.e., pores smaller than 2 nm) within the walls of the mesopores. It is well known that wall microporosity plays an important role in mesoporous silica prepared from P123 [35–36]. The value of *E*_F_ ≈ 5.0 GPa obtained from Equation 2 agrees indeed fairly well with the one found in SBA-15 (*E*_F_ ≈ 6 GPa) with a similar volume fraction of mesopores [27].

Now we try to estimate the maximum sorption-induced deformation ε and thus, the cantilever deflection, on a more fundamental basis. Gor and Neimark [13] introduced an analytical model based on the Derjaguin–Broekhoff–de Boer theory, which is able to derive the pressure *P* within a mesopore from a rigorous thermodynamic treatment of the liquid–solid and the liquid–gas interfaces involved. Knowing the pressure within the pores the resulting surface and bulk stresses in the solid pore walls can be calculated and, by using elasticity theory, the strains of the porous material can be obtained. This step is generally not a simple task as it depends critically on the geometry of the pore network. For cylindrical pores on a hexagonal pore lattice it was found recently that the pore lattice strain can be approximated by the radial strain within the wall of a cylindrical pore subjected to an internal pressure *P* [27,37]. Following [37], the strain within a thick cylinder with pore volume fraction 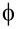, pore wall Young’s modulus 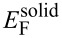, and corresponding Poisson’s ratio ν is given by

[3]
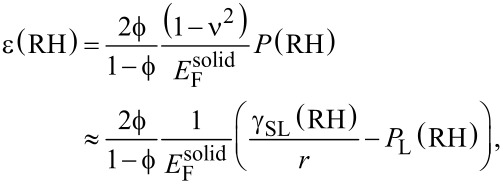


where *P*(RH) is the humidity-dependent pressure within the pore. To derive the right side of Equation 3, we have used the fact that ν is smaller than 0.2 for silica, and therefore (1 – ν^2^) ≈ 1. The first pressure term on the right hand side depends on the mean radius of curvature of the pores, *r*, and the absolute value of the solid–liquid interfacial energy, γ_SL_(RH)*,* which is determined by the amount of water adsorbed during successive pore filling [13,30,38]. In addition to the solid–liquid interfacial energy, both, the curved liquid–vapor interface during adsorption, and the formation of a hemispherical meniscus at the pore ends after capillary condensation lead to a Laplace pressure *P*_L_(RH). While the change of γ_SL_ gives rise to an increase of ε with increasing RH, *P*_L_ is opposite in sign. This leads to the often observed non-monotonous shape of the strain isotherms [13,27,37], with a sudden drop of the strain at capillary condensation. The reason, why there is no such strain drop seen in Figure 4 (and Figure 2) at the pressure of capillary condensation (RH ≈ 70%) is not fully clear. Theoretical calculations [13,39] and experiments with water in SBA-15 [40] show that this drop may be rather small for the adsorption branch. There should be, however, a clear increase in slope above capillary condensation, which is seen in both, the strain isotherm (Figure 4) and the cantilever deflection (Figure 2) at RH ≈ 70–80%.

To come to an estimate for the measured cantilever deflection, we assume for simplicity all mesopores being fully empty for the lowest RH (RH < 10%) and fully saturated with water without any menisci (meaning *P*_L_ = 0 in Equation 3) for the highest RH (RH ≈ 85%). Then, γ_SL_ = Δγ is the difference between the surface energy of silica and the interfacial energy of bulk silica fully wetted with water, which is known from literature (Table 1). If we make the additional assumption that the Young’s modulus of the porous silica has a quadratic dependence on its density, i.e., 
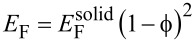
 [34] and insert the simplified Equation 3 into Equation 2 we obtain

[4]
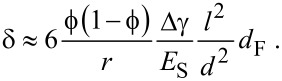


Most interestingly, within this model the cantilever deflection does not depend anymore on the elastic properties of the porous film, but only on the Young’s modulus of the silicon substrate, which is well known (Table 1). Hence, Equation 4 can be used to estimate the deflection δ from parameters derived from the nanostructure of the film and from the cantilever geometry only. Inserting the values summarized in Table 1 into Equation 4 yields a calculated deflection of δ = 86 nm, which is in fair agreement with the measured value of about 140 nm, given all the assumptions made to derive this result. The largest uncertainty comes certainly from the film thickness, which is not homogeneous within the cross section (see Figure 1b). As a final remark we note that the expression 
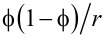
 in Equation 4 is closely related to the total volume specific surface area of the mesoporous material comprising the film. Hence, in the limit of thin compliant films as compared to the substrate, the deflection increases linearly with the specific surface area and with film thickness, and quadratically with the length/thickness ratio of the cantilever. In particular, it is noted that when a 1 μm thick substrate would be used instead of the 4.5 μm thick substrate of the present work, a deflection of roughly a factor 20 larger as compared to the current work can be expected.

## Conclusion

We have demonstrated the fabrication and humidity-induced deflection of a microcantilever based on a mesoporous silica/nonporous silicon bilayer. The principle of humidity-induced deformation of mesoporous silica is utilized to create a strain in the porous film, which leads to a reversible cantilever deflection of roughly 140 nm measured with an AFM. Grazing-incidence small-angle scattering (GISAXS) is used to determine nanostructural parameters of the pores such as their volume fraction and their mean curvature radius. Measurement of the RH-dependent pore lattice strain with GISAXS and comparison with the cantilever deflection using classical bilayer bending theory allows for estimating the Young’s modulus of the porous film. This is remarkable insofar, as the determination of elastic properties of highly porous thin films is quite important and not at all trivial [41–43]. We also develop a simple model to describe the cantilever deflection quantitatively. The model includes the determination of the sorption-induced strain from nanostructural parameters of the porous film, following the rigorous thermodynamic treatment of sorption-induced deformation introduced by Gor and Neimark [13]. It is shown that the maximum cantilever deflection at high RH depends on geometrical parameters (cantilever length and thickness of film and substrate) and on nanostructural parameters of the film (mean curvature and volume fraction). The only material-dependent properties entering are the Young’s modulus of the substrate and the change of the surface energy of the pore wall material with wetting, which for the silica–water interface is well known. Within certain limits, all the mentioned parameters may be varied to quantitatively control the deflection of the presented simple bilayer actuator in order to tailor its response to a humidity change. In combination with cantilever arrays, this concept could be used for complex, humidity-controlled switching operations.
